# A Review on Artificial Micro/Nanomotors for Cancer-Targeted Delivery, Diagnosis, and Therapy

**DOI:** 10.1007/s40820-019-0350-5

**Published:** 2019-12-30

**Authors:** Jiajia Wang, Renfeng Dong, Huiying Wu, Yuepeng Cai, Biye Ren

**Affiliations:** 1 School of Chemistry and Environment, Guangzhou Key Laboratory of Materials for Energy Conversion and Storage, Guangdong Provincial Engineering Technology Research Center for Materials for Energy Conversion and Storage, South China Normal University, Guangzhou, 510006 People’s Republic of China; 2 School of Materials Science and Engineering, South China University of Technology, Guangzhou, 510640 People’s Republic of China

**Keywords:** Micro/nanomotors, Cancer, Drug delivery, Diagnosis, Imaging

## Abstract

Micro/nanomotors have been extensively explored for efficient cancer diagnosis and therapy, as evidenced by significant breakthroughs in the design of micro/nanomotors-based intelligent and comprehensive biomedical platforms. Here, we demonstrate the recent advances of micro/nanomotors in the field of cancer-targeted delivery, diagnosis, and imaging-guided therapy, as well as the challenges and problems faced by micro/nanomotors in clinical applications. The outlook for the future development of micro/nanomotors toward clinical applications is also discussed. We hope to highlight these new advances in micro/nanomotors in the field of cancer diagnosis and therapy, with the ultimate goal of stimulating the successful exploration of intelligent micro/nanomotors for future clinical applications.

## Highlights

- Recent advances of micro/nanomotors in the field of cancer-targeted delivery, diagnosis, and imaging-guided therapy are summarized.
- Challenges and outlook for the future development of micro/nanomotors toward clinical applications are discussed.

## Graphical abstract

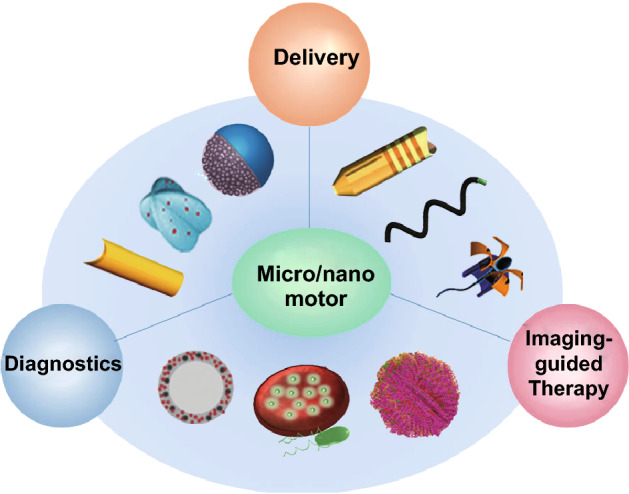

## Introduction

Cancers are emerging in an endless stream, leading to severe threat to human health [1]. To date, cancer treatment is still a worldwide problem and the eradication of cancers have been the lifelong pursuit of many scientists and physicians. Great efforts have been made to fight against cancers, and a large number of therapeutic approaches have been developed for clinical treatment of cancers, such as chemotherapy, gene therapy, radiotherapy, immunotherapy, and phototherapy [2]. However, it is still difficult to cure and eliminate cancer by relying solely on these techniques since the effectiveness of cancer treatment is strongly related to the stage at which the malignancy is detected. The tremendous progresses of nanotechnology and nanomaterials have paved new ways to address some pressing challenges in precise cancer diagnostics and therapy. Nanomaterials with intelligent properties have enabled exciting opportunities for solving the traditional complex problems that cannot be reached with conventional methods [3–6]. A variety of nanomaterials, such as magnetic nanobeads (MNs) [7], quantum dots (QDs) [8], upconverting nanomaterials (UCNPs) [9], hydrogel sheets [10], and gold nanoparticles (AuNPs) [11], have been rationally fabricated, leading to great revolution in nanomedicine. These nanomedicine platforms that allow for precise and remote manipulation of nanoscale objects in biological environments have enabled exciting opportunities for precise diagnostics and therapy of cancers.

In order to achieve precise treatment of cancer, it is required that the nanoparticles are distributed at a reasonable time and space at the target site, so they can be efficiently taken up and released by the cells, which requires a reasonable construction of the delivery system to avoid multiple obstacles to transport to the tumor site. Self-propelled micro/nanomotors, an emerging and powerful agent that is capable for effectively converting diverse energy sources into driving forces and autonomous movement [12–20], have gained considerable attention in the field of tumor diagnostics and treatment. Self-propelled micro/nanomotors not only inherit the excellent properties of micro/nanomaterials, such as high surface area and activity, but also demonstrate the distinct feature of autonomous motion capacity, which both results in highly efficient bioseparation and in precise delivery of imaging agents or drugs to the subcellular target in tumors [21–30]. The active propulsion of these motors plays a key role for their biomedical applications. Therefore, efficient energy harvesting is crucial in the design of micro/nanomotors-based delivery systems. Generally, fuel-dependent micro/nanomotors used in biological systems should be propelled by non-biotoxic or biocompatible fuels. Fuel-free micro/nanomotors, powered by different types of energy sources such as magnetic, electric field, or acoustic, endow more flexibility in biological applications. In addition to the characteristics of active motion, the micro/nanomotors should generally exhibit the following features, such as: (1) miniaturized size and proper morphology, (2) easy surface functionalization, (3) good biological compatibility, and (4) low toxicity chemical composition.

To meet these demands of efficient energy harvesting and conversion, a wide variety of fabrication strategies have been developed, such as metallic thin films deposition, template-assistant metal electroplating, rolling-up methods, and self-assembly techniques [31, 32]. With these techniques, a broad array of micro/nanomotors have been fabricated, such as nanowires [33–35], microtubular microrockets [36–38], Janus microspheres [39–41], and supermolecule-based nanomotors [42–44]. They can efficiently be moved by harnessing various fuels and various driving modes [45–47]. Coincidentally, cancer cells have been found to generate oxidative stress by producing an elevated level of hydrogen peroxide (H_2_O_2_) and exhibit acidic pH levels. This will provide an energy source for micro/nanomotors that harness local H_2_O_2_ or H^+^ to enable propulsion with incomparable advantages for in situ precise cancer target and penetrate into deep tumor tissues beyond regular diffusion limits [48]. In addition, synthetic micro/nanomotors designed with increasing ability to enable fuel-free operation have also gained significant attention. For example, ultrasound-driven nanowires that harness energy to generate an asymmetric pressure gradient for propulsion were able to target tumors in an active targeting manner [49]. Moreover, the active poration of cell membranes by micro/nanomotors has also been reported. Wang et al. reported an ultrasound-driven gold-nanoshell-functionalized polymer multilayer tubular nanoswimmer that can photomechanically perforate the membrane of a cancer cell by assistance of near-infrared (NIR) light [50]. Such nanoswimmers are capable of actively targeting a single cell and opening the cell membrane, which could be utilized for intracellular drug delivery, artificial insemination, and subcellular surgery processes. More importantly, micro/nanomotors can be combined with other nanomaterials such as QDs or UCNPs, which could enable the integration of cancer diagnosis and treatment on these nanoscale platforms [51, 52]. Collectively, these intriguing advantages of creative micro/nanomotors have remarkable potential for precise treatment of cancers.

**Fig. 1:**
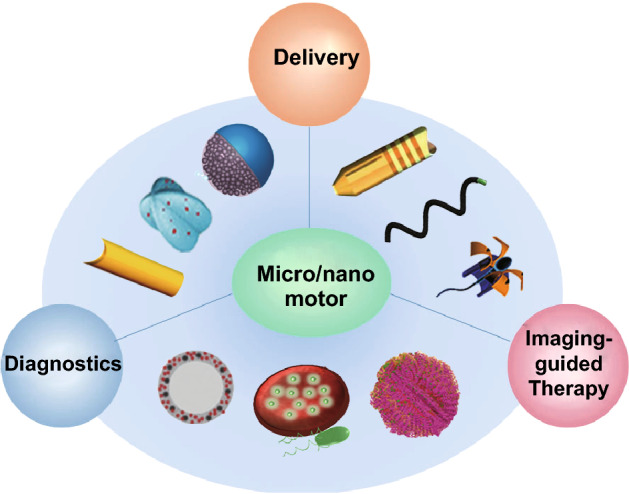
Schematic illustration of micro/nanomotors for cancer diagnosis and therapy

The utilization of the unique self-propulsion of micro/nanomotors for addressing challenging issues raised by cancer research has promoted the development of novel diagnosis method, imaging probes, intracellular delivery vehicles, and multifunctional dynamic therapies. This review article is mainly focused on the tremendous inspiration and opportunities offered by micro/nanomotors for potential use in the field of cancer diagnostics and treatment (Fig. 1). Firstly, we present the progress of micro/nanomotors in the fields of cancer-targeted delivery, such as drug delivery, interfering RNA delivery. Secondly, we illustrate the utilization of micro/nanomotors for cancer diagnostics, ranging from isolation of circulating tumor cells to detection of cancer-related biomarkers such as protein and microRNA and image-guided photothermal tumor therapy. Finally, we comment on the current problems and challenges faced by micro/nanomotors in tumor detection and treatment and present an outlook for the future development of micro/nanomotors toward clinical applications. All in all, the clinical application of micro/nanomotors in the field of cancer diagnosis and therapy still has a long way to go. We hope this review can greatly promote the development of future intelligent micro/nanomotors toward precise cancer treatment and provide a new outlook for overcoming some pressing challenges in cancer treatment.

## Cancer Cell-Targeted Delivery

Currently, traditional delivery systems rely solely on circulation of body fluids and lack sufficient penetration and targeting, which makes it difficult for drugs to penetrate tissue and the interior of the lesion. This seriously affects the therapeutic effect of the drug. Anticancer agents such as proteins, siRNAs, and plasmids exhibit excellent biological activity and therapeutic effects in the cytoplasm, and their emergence has brought new choices for advancing the treatment of cancer [53]. However, effectively delivering these agents to the cytoplasm while still ensuring their activity is still very difficult. Transfection techniques can be used to deliver them to cells *via* some biological vectors such as viral vectors or liposome complexes, but the use of biological vectors could introduce an uncontrolled risk of delivery. In order to deliver the drug precisely to the target disease site, the drug carrier needs to have some unique capabilities, including propulsion, cargo and release, and penetration. Given these challenges in drug delivery systems, the advent of micro/nanomotors offers a new and attractive option for drug delivery, thereby increasing the therapeutic effect and reducing the systemic side effects of highly toxic drugs [54–56]. Here, the self-propelled micro/nanomotors provide the carriers with continuous driving power to help them transport across biological tissues. In addition, the direction of the motor can also be adjusted to prompt cell targeting and internalization, so as to enhance the controllability and adjustability of drug delivery system [57–59]. Compared to passive drug carriers, the self-propulsion abilities of these micro/nanomotors have unique advantages in drug delivery, which may bring distinct improvements for efficient drug delivery.

### Extracellular Delivery System

**Fig. 2:**
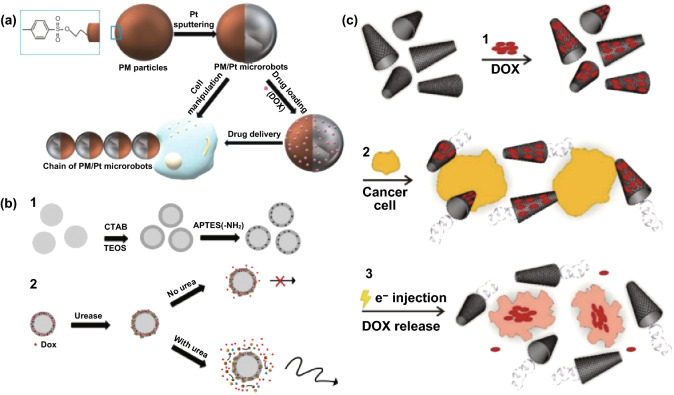
**a** Schematic illustration of the fabrication of PM/Pt microrobots for cell manipulation, anticancer doxorubicin (DOX) drug loading, and delivery. Adapted from Ref. [61] with permission, copyright Wiley Ltd., 2018. **b** Enzyme-powered nanobots enhance anticancer drug delivery. Adapted from Ref. [62] with permission, copyright Wiley Ltd., 2017. **c** An overview on the loading and release of doxorubicin on/from n-rGO/Pt micromachines. (1) Physical adsorption of doxorubicin on the outer layer of micromachine (n-rGO). (2) Bubble-propelled swimming of micromachines loaded with doxorubicin in the presence of cancer cells (yellow). (3) The release of doxorubicin upon applying an electrical potential and delivering the drugs to the cancer cells. Adapted from Ref. [63] with permission, copyright Wiley Ltd., 2019. (Color figure online)

Numerous studies have been made in the fabrication of micro/nanomotors that meet the requirements of ideal drug delivery vehicles and promote the development of micro/nanomotors-based drug delivery systems. Initially, a number of synthetic micro/nanomotors have been designed to promote cancer cell targeting and further promote drug release (extracellular delivery system). It has been found that cancer cells produce oxidative stress by producing high levels of H_2_O_2_ [60]. Therefore, a series of synthetic micro/nanomotors have been designed to harness local H_2_O_2_ as an energy source for propulsion to demonstrate the delivery performance. For example, Villa et al. [61] reported multifunctional superparamagnetic/catalytic microrobots (PM/Pt microrobots) for cell manipulation and anticancer drug delivery (Fig. 2a). These micromachines contain a superparamagnetic core, allowing them to assemble as micromotor chains and navigate by an external magnetic field. The Pt hemisphere of Janus microsphere enables catalytic self-propulsion, while tosyl groups enable the motors to manipulate cells and to deliver bioactive molecules. After loading them with the anticancer water-soluble drug doxorubicin (Dox), these microrobots are able to capture breast cancer cells and simultaneously release the drug into the cancer cells by diffusion. Such versatile device simplifies the applicability of nanomotors toward diverse biomedical applications. The exploit of biocompatible enzymes to power micro/nanomotors has opened new avenues for active delivery of specific drugs to the site of interest. Compared to other catalytic motors, enzyme-powered micromotors possess several advantages, such as bioavailable and biocompatible, which are more suitable for biological applications. Hortelão presented enzyme-powered nanomotors for enhancing anticancer drug delivery (Fig. 2b) [62]. The nanomotors comprised of a solid silica core which was coated with urease enzymes and a mesoporous silica shell which provided high loading capacity of Dox. Furthermore, the urease enzymes-modified nanomotors convert chemical energy into mechanical work even in ionic media (phosphate buffer saline buffer solution), which demonstrated their potential use in biomedical applications. The nanomotors-based Dox-loaded system obtained an enhanced anticancer efficiency toward HeLa cells, which arises from a synergistic effect of the enhanced drug release and the ammonia produced at high concentrations of urea substrate. Increased drug delivery efficiencies achieved by these nanomotors may have potential for use in future biomedical applications. In addition to the above applications, micro/nanomotors have also been made into tubular structures to harness the chemical energy of H_2_O_2_ (Fig. 2c) [63]. In this design, reduced nanographene oxide (n-rGO)/platinum (Pt) micromachines were fabricated for efficient drug delivery. Notably, the n-rGO/Pt micromachines showed fast speed even in very low concentration of H_2_O_2_ and high loading efficiency of Dox molecules. In addition, the authors also integrated the nanocarriers with electrochemical stimulus, which lead to drug release in a specific location in only a few second. Coupling the powerful self-propulsion and high loading capacity of n-rGO/Pt micromachines with the introduced ultra-fast drug release mechanism, the self-propelled machine provides an important step toward the realization of advanced drug delivery systems.

### Intracellular Delivery System

Following the initial success of micro/nanomotor-based delivery systems, research in the field of intracellular delivery has advanced further. Currently, a series of micro/nanomotors, such as H_2_O_2_-powered microrobots, enzyme-powered micromotors, magnetic, or ultrasound field driven-micro/nanomotors, have been demonstrated for efficient delivery. Importantly, in order to achieve efficient delivery toward cancer cells and considerable intracellular release of payloads, the micro/nanomotors must be rationally synthesized to overcome multiple transport barriers. For example, the characteristics such as size, charge, shape, and surface chemistry should be comprehensively considered to facilitate the efficient delivery of drugs or payloads to the subcellular target in tumors. In addition, for in vivo delivery, the interaction between the micro/nanomotors and various tissues (e.g., liver, kidney, spleen, brain, and tumor), the nonspecific interactions with healthy cells as well as the clearance efficiency of the micro/nanomotors are also should be considered.

**Fig. 3:**
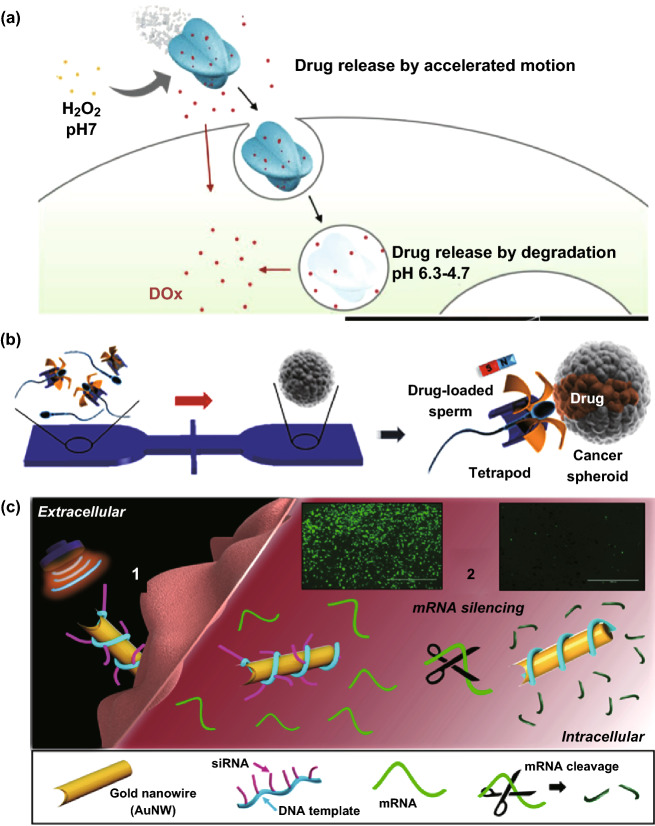
**a** Superassembled biocatalytic porous framework micromotors (cat-β@ZIF) with reversible and sensitive pH-speed regulation for drug delivery. Adapted from Ref. [64] with permission, copyright Wiley Ltd., 2019. **b** Schematic of the microfluidic chip for drug-loaded sperm transport and delivery. Adapted from Ref. [65] with permission, copyright American Chemical Society, 2017. Notice: further permissions related to this material excerpted should be directed to the ACS. **c** Acoustically propelled nanomotors for intracellular siRNA delivery. Adapted from Ref. [66] with permission, copyright American Chemical Society, 2016

Some work has been reported on the use of micro/nanomotor to penetrate cell membranes and achieve efficient delivery of various therapeutic compounds into cells. For example, Gao et al. [64] developed succinylated β-lactoglobulin and catalase-assembled biocatalytic micromotors for enhanced intracellular drug release (Fig. 3a). The succinylated β-lactoglobulin in the micromotors has a pH-responsive function, which could regulate the access of H_2_O_2_ fuel into the micromotor. Therefore, the state of the micromotors can be switched between “on” and “off” by adjusting the pH of the outside world. At pH 7, the biological H_2_O_2_ was able to access catalase, leading to autonomous movement of the micromotors, followed by cellular uptake and prolonged retention of the particles in acidic compartments (pH 6.3–4.7). Here, the micromotors were degraded, leading to the release of drugs from the micromotor. Notably, this study reported the first chemically driven motors with reversible pH-responsive motility.

Additionally, fuel-free micro/nanomotor driven by external power, such as magnetic or ultrasound fields, also shows great potential for intracellular precise delivery. For a biocompatible delivery system, a magnetic-driven sperm micromotor is presented as a targeted drug delivery (Fig. 3b) [65]. This system is demonstrated to be an efficient drug delivery vehicle by first loading a motile sperm cell with doxorubicin hydrochloride. Under external magnetic field, the sperm-driven micromotor can be guided to the tumor spheroid, free the sperm cell as well as release the drug. Compared to purely synthetic micromotors, the sperm-hybrid micromotor can encapsulate high concentrations of doxorubicin hydrochloride inside the sperm membrane. Also, the ability of sperms to fuse with somatic cells represents a unique property to deliver the drug locally into cancer cells through sperm cell membrane fusion. These sperm-hybrid micromotors not only have potential applicability for gynecologic cancer treatment, but also for the therapy of other diseases in the female reproductive tract.

Ultrasound (frequencies above the audible range of humans; N20 kHz) can be focused on very small areas due to its wavelength in the order of millimeters. It has been employed for both enhancing drug delivery and improving drug activity for over two decades. For example, the unique ability of ultrasound-propelled gold nanowires for cell penetration was also applied to siRNA delivery (Fig. 3c) [66]. In this design, nanowires were wrapped with rolling circle amplification (RCA) DNA structures hybridized with green fluorescence protein-targeted siRNAs. The critical role played by ultrasound propulsion was reflected by the comparative studies between propelled and static motors. The results showed a 94% silencing on HEK293 and MCF-7 cells after treatment for a few minutes, approximately 13-fold improvement in the silencing response as compared to the static nanomotors. Motor optimization studies implied that the nanomotors pierced the cell membrane and continued to move rapidly inside the cells, which together led to a high gene-silencing efficiency.

**Fig. 4:**
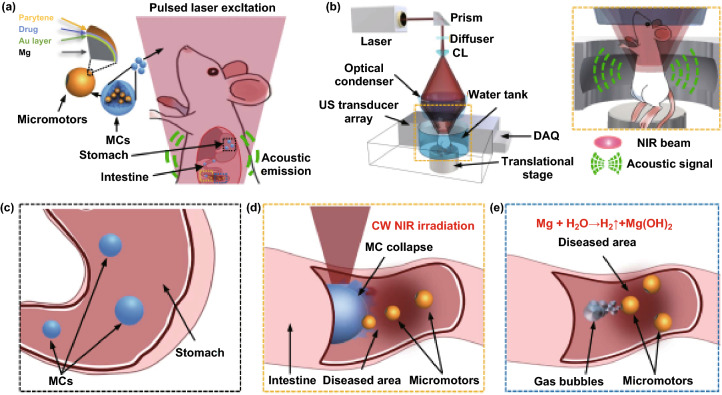
Schematic of PAMR in vivo. **a** Schematic of the PAMR in the GI tract. The MCs are administered into the mouse. NIR illumination facilitates the real-time PA imaging of the MCs and subsequently triggers the propulsion of the micromotors in targeted areas of the GI tract. **b** Schematic of PACT of the MCs in the GI tract in vivo. The mouse was kept in the water tank surrounded by an elevationally focused ultrasound transducer array. NIR side illumination onto the mouse generated PA signals, which were subsequently received by the transducer array. (Inset) Enlarged view of the yellow-dashed box region, illustrating the confocal design of light delivery and PA detection. US, ultrasound; CL, conical lens; DAQ, data acquisition system. **c** Enteric coating prevents the decomposition of MCs in the stomach. **d** External CW NIR irradiation induced the phase transition and subsequent collapse of the MCs on demand in the targeted areas and activated the movement of the micromotors upon unwrapping from the capsule.** e** Active propulsion of the micromotors promoted retention and cargo delivery efficiency in intestines. Adapted from Ref. [67] with permission, copyright American Association for the Advancement of Science, 2019

Notably, a photoacoustic computed tomography-guided microrobotic system which enables deep imaging and precise control in vivo has been reported [67]. The micromotors are wrapped in microcapsules and thus protected from gastric acid. The migration of microcapsules toward the lesion area can be observed in real-time in vivo by PACT. Near-infrared light irradiation toward targeted areas induces disintegration of the capsules and triggers the propulsion of the micromotors, which can effectively extend the residence time of the drug (Fig. 4). Due to the merits of high spatio-temporal resolution, non-invasiveness, molecular contrast, and deep penetration, the PACT-based microrobotic system provides an attractive tool for targeted delivery in vivo. Considering the tremendous progress made recently in the development of micro/nanorobots and their uses toward in vivo delivery, these micro/nanorobots are expected to become powerful active transport vehicles that may enable a variety of therapeutic applications that are otherwise difficult to achieve through the existing passive delivery systems.

## Cancer Diagnostics

The effectiveness of cancer therapy is directly related to the accuracy of cancer diagnosis, which depends heavily on the stage at which the malignancy is detected. Therefore, early screening of tumors has become desirable and has played a key role in improving the treatment rate of cancers, especially for breast and cervical cancer in women, and colorectal and prostate cancer in men [68]. However, most solid tumors are not detectable unless they reach approximately 1 cm in diameter, at which point most tumors have already metastasized. In clinical practice, conventional and common cancer diagnostics techniques mainly include bioanalytical assays of bodily fluids, medical imaging, and tissue biopsy [69]. Most of these methods currently suffer from insufficient sensitivity and/or specificity to detect most types of cancers at this critical early stage, which may be in part why cancers are generally detected with delay and are often diagnosed at an advanced stage, when it is least curable. Due to the particularity and complexity of tumor, the diagnosis can be achieved by directly detecting the cancer cells or cancer tissue [70]. Other diagnostic methods can also be achieved indirectly by detecting tumor-related biomarkers such as protein or microRNA [71]. For example, prostate-specific antigen (PSA) has been used as an effective indicator of prostate cancer so as to improve diagnostic accuracy of prostate cancer [72], and the express level of alpha-fetoprotein (AFP) is an important biomarker for the diagnosis of primary liver cancer [73]. In cancer detection, as targets usually exist within complex biological matrices, their highly efficient identification and isolation are crucial and necessary steps for accurate cancer diagnosis. The emergence of micro/nanomotors provides powerful ways for efficient diagnosis of tumor cells and markers. As mentioned before, due to their active motion characteristics, the functionalized micro/nanomotors can facilitate the mixing of bioprobes with targets, which greatly improve the target binding efficiency and further enhance biodetection sensitivity and efficiency. Furthermore, the micro/nanomotors are easy to conjugate with biomolecules. Great progress has been made by using micro/nanomotors in the detection of various targets such as ions, protein, and RNA that have been widely used in bioassays [74]. We will mainly introduce the application of micro/nanomotors in the detection of cancer cells, cancer-related biomarkers, and other substances relative to the occurrence of cancer.

### Cancer Cells Detection

For the detection of cancer cells, efficient isolation and enrichment of cancer cells are key and necessary steps for accurate detection due to the complex biological matrices in which these cells exist. With the development of nanotechnology and nanomaterials, a series of methods for tumor cell separation have also been developed. Among many nanomaterials, magnetic nanospheres, with excellent superparamagnetic properties and isolation performance, are a preferred choice for isolation and detection of tumor cells. For example, quick-response magnetic nanospheres have been synthesized by a layer-by-layer assembly method and further modified with the anti-EpCAM antibody, and the obtained immunomagnetic nanospheres can be used for efficient and fast capture of tumor cells in whole blood, with an efficiency of more than 94% in only 5 min [75]. Commercial automated immunomagnetic enrichment technology for CTC detection, known as CellSearch, has been approved by the US Food and Drug Administration (FDA). In this system, the magnetic nanospheres (120–200 nm) were modified with anti-EpCAM antibody and biotin analogue, and then, the functionalized magnetic nanospheres were used to capture tumor cells [76–78]. The technology shows high sensitivity and accuracy. However, the capture based on magnetic nanospheres can only be achieved by the diffusion of fluids. Due to the high viscosity of blood and tissue, it is also necessary to use some mixing means such as shaker and mediation to improve the capture efficiency, which not only made the operation procedure complicated, but also induced cell loss. In addition, in the in situ detection of tumor cells, the magnetic nanospheres are difficult to reach where the body fluid circulation cannot reach. Therefore, materials with special functions are urgently needed to solve this problem. The advent of micro/nanomotors with autonomous operating characteristics has brought dawn to the efficient separation of tumor cells.

Along with rapid advances in synthesis technology, specific micro/nanomotors have been efficiently fabricated for efficient capture and separation of targets. In an early study, the researchers prepared a nanowire motor to further integrate the magnetic nanoparticles into the motor, making the motor to have the characteristics of loading, transporting, and releasing [79, 80]. In addition to this, a chemical reaction-based loading strategy has also been reported. Here, the target is attached to a motor via a photocleavable o-nitrobenzyl linker, which undergoes photolysis and unloading of the cargo under ultraviolet irradiation [81]. Helical micromotors are also used to manipulate individual cells. In this work, the micromotor was first turned to human B lymphocytes; after contact, the motor assembled and continued to move toward the target position. Upon arrival, the motor was dismantled [82]. Moreover, the highly specific detection and isolation capabilities of such micro/nanomotors make them particularly attractive in building sensing microchip devices. Several groups have explored the incorporation of micro/nanomotors within fluidic chip. Sensing, pickup, and analysis along predetermined paths within the channel networks were achieved, thus simplifying the common biomedical analysis procedure [83]. Collectively, these previous studies paved the way for the use of micro/nanomotors in the separation and detection of tumor cells through the “transportation and release” of various targets, including the use of motors to reach and isolate tumor cells.

**Fig. 5:**
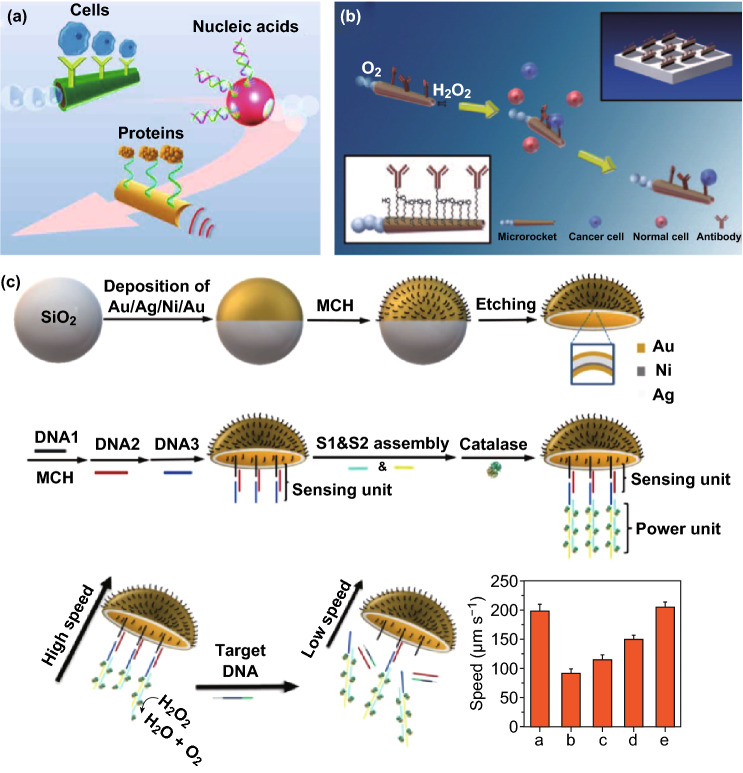
Strategies and examples of micro/nanomotors for detection. **a** Functionalization of micro/nanorobot with different bioreceptors toward biosensing of cancer target, including cells, proteins, and nucleic acids. Adapted from Ref. [28] with permission, copyright from American Association for the Advancement of Science, 2017. **b** Micromotors for capture and isolation of cancer cells. Adapted from Ref. [84] with permission, copyright Wiley Ltd., 2011. **c** Schematic diagram of micromotor-based DNA sensing. Adapted from Ref. [85] with permission, copyright American Chemical Society, 2019

Inspired by the excellent performance of micro/nanomotors, there have been a series of studies on the use of micro/nanomotors to achieve cancer diagnosis (Fig. 5a), ranging from cells, nucleic acids, and proteins, which provide potential options for accurate cancer diagnosis [28]. A hollow-shape micromotor consisting of a three-layer rolled metal plate (inner platinum, middle iron and gold outer) (Fig. 5b) was fabricated for the efficient isolation and detection of circulating tumor cells (CTCs) [84]. The inner layer of platinum allows the catalyzation of peroxide to oxygen and water, which could provide efficient power for the active movement of the micromotor. The middle iron layer endows the micromotor with superior magnetic separation ability. The gold layer outside provides easy and convenient surface modification of antibodies or molecules that are attuned to proteins overexpressed on the cell surface. The unique design allows the micromotor to have a large surface to volume ratio, a relatively high speed in the diluted serum (about 85 μm s^−1^), and the ability to pull larger cancer cells. For the further detection application, the micromotor was functionalized with antibodies that allows it to specifically identify target cells while bypassing nontarget cells. The obtained immunomicromotors were able to successfully pick up and transport CTCs with a high efficiency. It is worth mentioning that the cargo of the CTC has negligible impact on the speed of the micromotor since the speed drops slightly to ~ 80 μm s^−1^ in the same medium. This study provides a potential horizon for future efficient cancer cells isolation in biological fluids with high ionic strength and viscosity.

### Cancer-Related Biomarkers Detection

Apart from the detection of cancer cells, biomolecule biomarkers (such as proteins and genes) have also been widely used for cancer diagnosis in clinical settings. The detection of cancer-related biomarkers can reflect the existence and growth of tumors to a certain extent, which has great guiding significance for the early detection of tumors and the development of treatment plans. Since the nanomotor has the characteristics of active motion, the speed change of the motor interacting with the target can be used as a signal for detecting the target. For example, Zhang et al. [85] proposed a jellyfish-like micromotor for the detection of target DNA (Fig. 5c). The concave surface of the micromotor was assembled with a sandwich DNA hybridization. In the presence of target DNA, the catalase was released from micromotors, resulting in the reduction in motion speed. Thus, just by recoding the speed of the micromotor, the detection of DNA can be realized, which is simple, cheap, and fast.

In addition, miRNAs detection also shows great significance in clinical cancer diagnosis. It has been reported that the expression level of miRNAs is related to cancer initiation, tumor stage, as well as tumor response to treatments [86]. Therefore, the real-time monitoring of the changes of miRNAs is meaningful for cancer diagnosis and prognosis. Nevertheless, the detection of miRNAs is very challenge since their intrinsic small size with a length of 17–25 nucleotides, sequence homology, and low relative concentration in total RNA samples. Conventional detection methods such as quantitative reverse transcription polymerase chain reaction (qRT-PCR) and Northern blotting usually require multifarious operation steps and specific instruments [87]. Nanomaterials with attractive properties provide new choices for miRNAs analysis. Novel strategies based on nanomaterials such as magnetic nanoparticles [88], QDs [89], and gold nanoparticles [90] have also been developed with increased sensitivity and convenient. However, these methods still cannot meet the requirement of in situ detection of miRNAs in cells, especially in single cells.

**Fig. 6:**
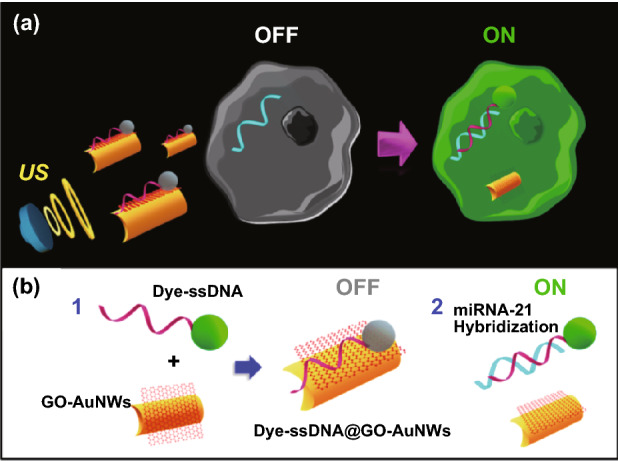
**a** Intracellular detection of miRNA in intact cancer cells using ultrasound (US)–propelled nanomotors. **b** the “OFF–ON” fluorescent switching system for the specific detection of miRNA-21 in intact cancer cells, and steps involved immobilization and quenching of the dye fluorescence. Adapted from Ref. [91] with permission, copyright American Chemical Society, 2015

The active micro/nanomotors provide an excellent and powerful option for the detection of trace amounts of targets. The sensing strategy based on micro/nanomotors mainly relies on their motility, and by further modifying them with different biological receptors or molecules on its surface, they can be endowed with recognition ability or specific functions. Then, the detection can be realized by detecting the change of the signal after identifying the target. Efforts have also been directed to the use of micro/nanomotors for miRNA sensing, and a series of strategy have been constructed. For example, methods for achieving intracellular miRNA sensing by exploiting the internalization and movement of nanomotors within cells have been realized. Based on the use of an ultrasound-propelled nanomotor functionalized with single-stranded DNA (ssDNA), Esteban-Fernández de Ávila et al. [91] introduced an attractive intracellular “off-on” fluorescence strategy for detecting the endogenous content of target microRNA-21 (miRNA-21) (Fig. 6). The presence of the target miRNA resulted in displacement of the dye-ssDNA probe from the surface and a fast fluorescence recovery of the quenched dye-labeled specific ssDNA probe. Such nanomotor-based biosensing approach leads to new ways to monitor miRNA expression at the single-cell level, promoting the investigation of miRNA in clinical applications.

## Imaging-Guided Therapy

Early diagnosis of tumors has great medical significance. Apart from the detection of tumor cells and biomarkers, cancers can also be diagnosed through medical imaging. Efficient imaging agents can provide precise tumor localization, offer effective early diagnosis and treatment monitoring for precise cancer treatment, and provide considerable guidance for intelligent cancer therapy. Currently, a great number of biomedical imaging techniques, such as magnetic resonance imaging (MRI), computer tomography (CT), and positron emission tomography (PET), have been developed, which have improved the spatial resolution of cancer imaging [92]. Concurrently, the progress of nanomaterials with unique properties has also become interesting prospects for precise cancer imaging. Among them, MRI and optical fluorescence imaging are the most value medical applications due to their non-invasiveness and non-aviation. Nanomaterials, such as QDs [93] and magnetic nanocomposites [94], have shown great application advantages for optical fluorescence imaging or MRI. For example, Xie et al. [95] prepared 4-methylcatechol modified MNPs, which could be directly conjugated with a peptide, c(RGDyK). The modified MNPs can be used for tumor cells imaging. Although it is highly promising to promote cancer treatment, conventional delivery of these imaging agents to the tumor site relies on random diffusion and systemic circulation, which hinders imaging of deep tumor tissue and results in low sensitivity of imaging. Self-propelled micro/nanomotors, which are able to self-drive by engaging the motion driven by in situ energy conversion, have recently attracted intense attention in the cancer imaging field. The combination of micro/nanomotors with other nanomaterials could help them transport across biological tissues, prompt targeting and internalization, so as to enhance the precision of imaging. Furthermore, micro/nanomotors with photothermal effects can also be used for imaging and therapeutic treatment.

### Cancer Cells Imaging

**Fig. 7:**
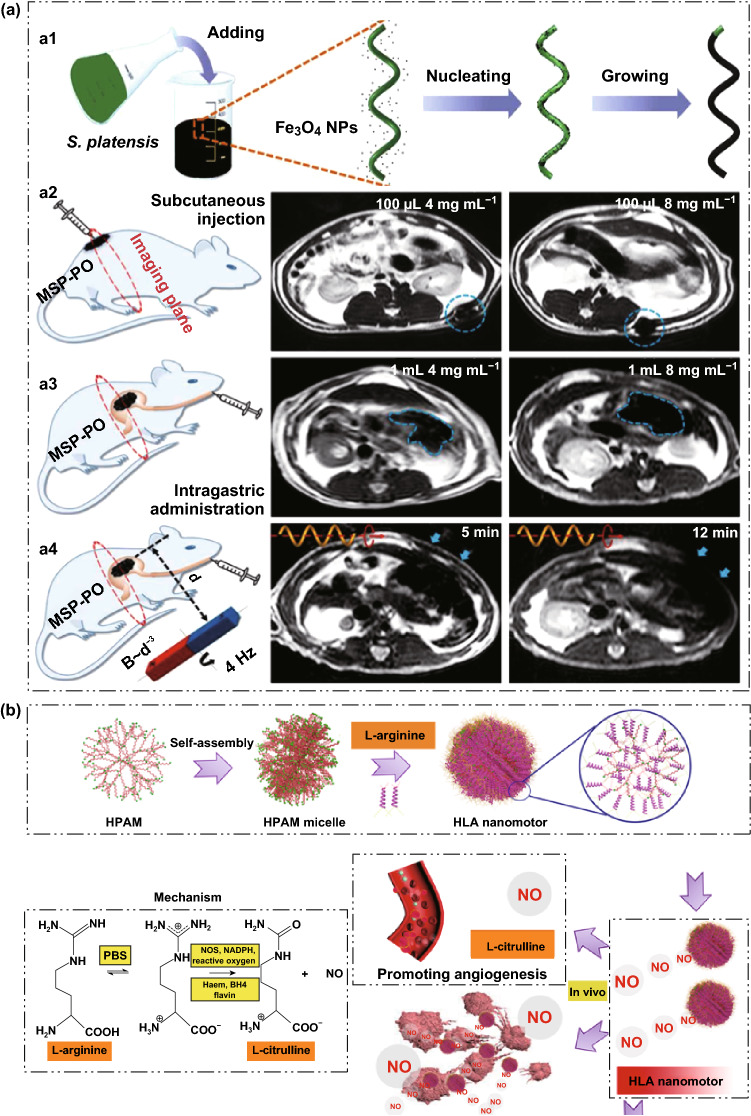
**a** (**a1**) Schematic of the dip-coating process of S. Platensis in a suspension of Fe_3_O_4_ NPs. **a2** MSP swarm of two different concentrations inside the subcutaneous tissues. **a3** MSP swarm of two different concentrations inside the stomachs. **a4** MSP swarm with the same concentration but subject to actuation and steering (with a rotating magnetic field) of different time periods before MR imaging across the rat’s stomach. Adapted from [96] with permission, copyright from American Association for the Advancement of Science, 2017. **b** Schematic illustration of the formation of zwitterion-based nanomotor and the NO generation principle. Adapted from Ref. [97] with permission, copyright American Chemical Society, 2019

Magnetic microrobots have been shown to be very useful for magnetic resonance imaging. These magnetic microrobots can be remotely controlled to propel in complex biological fluids with high precision by using magnetic fields. Their potential for controlling navigation in hard-to-reach cavities of the human body makes them promising miniaturized robotic tools to diagnose and treat diseases in a minimally invasive manner. For example, Yan et al. [96] have fabricated biohybrid magnetic robots from spirulina microalgae for being endowed with multifunctional capabilities by integrating desired structural and functional attributes from a biological matrix and an engineered coating (Fig. 7a). The microalgae allowed in vivo fluorescence imaging and remote diagnostic sensing without the need for any surface modification. Furthermore, in vivo magnetic resonance imaging tracked a swarm of microswimmers inside rodent stomachs, a deep organ where fluorescence-based imaging ceased to work because of its penetration limitation. Meanwhile, the microswimmers were able to degrade and exhibited selective cytotoxicity to cancer cell lines, subject to the thickness of the Fe_3_O_4_ coating, which could be tailored *via* the dip-coating process. These biohybrid microrobots represent a microrobotic platform that could be further developed for in vivo imaging-guided therapy and are a proof of concept for the engineering of multifunctional microrobotic and nanorobotic devices. Interestingly, in contrast to most nanomotors based on H_2_ or CO_2_, NO-expelled hyperbranched polyamide/l-arginine (HLA) nanomotors have been proposed, which utilize NO-producing endogenous biochemical reactions as their driving force (Fig. 7b) [97]. The produced NO also has beneficial effects, including promoting reendothelialization and anticancer effects. In addition, the HLA nanomotors are fluorescent, which is a concept that can be applied to monitor the movement of self-imaging nanomotors in vivo for future biological applications for the treatment of various diseases.

**Fig. 8:**
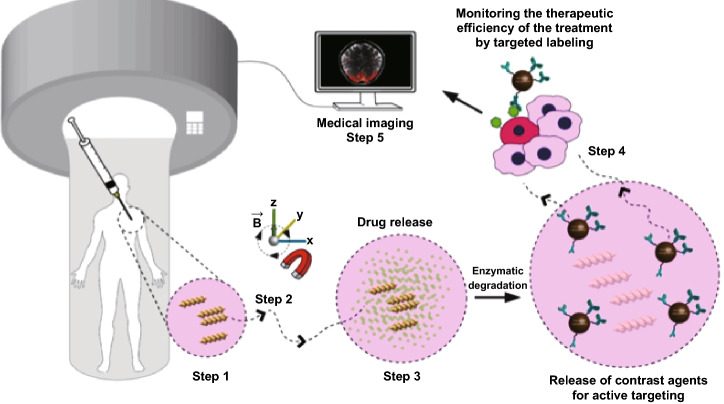
3D-printed biodegradable microswimmer for theranostic cargo delivery and envisioned theranostic application scenario of the 3D-printed biodegradable microswimmer. Adapted from Ref. [98] with permission, copyright from American Chemical Society, 2019. Notice: further permissions related to this material excerpted should be directed to the ACS

3D-printed biodegradable microswimmer has also been used for precise delivery. Ceylan et al. [98] introduced a 3D-printed biodegradable double-helical microswimmer using two-photon polymerization from a magnetic precursor suspension comprised from gelatin methacryloyl and biofunctionalized superparamagnetic iron oxide nanoparticles (Fig. 8). This double-helical microswimmer can achieve volumetric cargo loading and swimming capabilities under rotational magnetic fields. It is worth mentioning that the 3D-printed microswimmer could be gradually degraded to solubilized non-toxic products by matrix metalloproteinase-2 (MMP-2) enzyme at normal physiological concentrations. After full degradation, the drug carried by the microswimmer can be completely released into the given microenvironment. In addition to loading drugs, anti-ErbB 2 antibody-tagged magnetic nanoparticles were used as an example to demonstrate the delivery efficiency of microswimmers. The labeling of SKBR3 breast cancer cells in vitro imaging demonstrates the potential of microswimmer-based therapeutic delivery in future medical operation.

### Cancer Cells Phototherapy

**Fig. 9:**
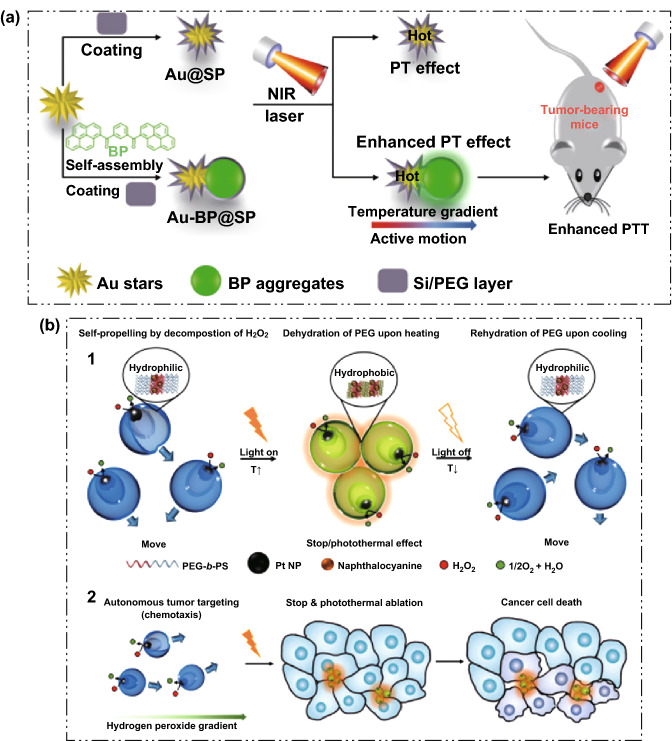
**a** Schematic illustration of a self-propulsion Au-BP@SP Janus nanoparticle for cancer cell treatment under NIR laser irradiation. Adapted from Ref. [103] with permission, copyright Wiley Ltd., 2016. **b** Light-guided nanomotor systems for autonomous photothermal cancer therapy. (1) Schematic representation of a light-guided nanomotor system using PEG44-b-PS141/naphthalocyanine (NC) and Pt nanoparticles (Pt NPs) powered by the conversion of hydrogen peroxide (H_2_O_2_). (2) Schematic illustration for the autonomous photothermal ablation of cancer cells using the nanomotors. Adapted from Ref. [104] with permission, copyright American Chemical Society, 2017

Micro/nanomotors-based phototherapy methods are expected to promote the development of efficient, specific, and personalized nanomedicine. Phototherapy includes photodynamic therapy (PDT) and photothermal therapy (PTT). PDT is mainly achieved by reactive oxygen species-mediated cell damage produced by the activation of photosensitizers [99, 100]. PTT mainly relies on the absorbent that can effectively convert the light energy of the NIR region (700–950 nm) into heat energy, which damages the tumor cells through high temperature [101, 102]. The application of micro/nanomotor in phototherapy has several advantages. For example, the micro/nanomotors have the natural advantage of integrated nanomaterials, which can accumulate in the tumor site through the EPR effect; additionally, the micro/nanomotors have the characteristics of active motion behavior, which can efficiently pass through the biological barrier and improve the efficiency of targeting tumor cells. At present, some materials with excellent photothermal effects have been used to prepare micro/nanomotors, which are expected to be applied to cancer imaging and photothermal therapy. Among them, gold nanoparticles have received extensive attention due to their excellent photothermal characteristics. For example, Yang et al. [103] have prepared Au-BP7@SP Janus nanohybrids which show thermophoresis with active motion and enhanced PT effect (Fig. 9a). Under NIR irradiation, the active motion of Au-BP7@SP Janus nanohybrids can effectively enhance the temperature of the treated cells, by converting the kinetic energy to thermal energy, further killing the cancer cells within 2 days. The Au-BP@SP nanohybrids can effectively locate the tumor site without affecting the adjacent normal tissue, thereby greatly improving the therapeutic effect and reducing adverse side effects. This study suggested that the active motion of Janus nanohybrids contributed to PTT of cancer cells and opened a new avenue to cancer treatment by using self-propelling Janus nanohybrids.

NIR light-driven stomatocyte nanomotors have also been fabricated for active photothermal cancer therapy. For example, Yang et al. have p prepared PEG44-b-PS141 nanomotor encapsulated with Cu (II) 5,9,14,18,23,27,32,36-octabutoxy-2,3-naphthalocyanine (NC) and Pt NPs, which can be used as a strong NIR light absorber and an engine and catalytic decomposer of H_2_O_2_, respectively [104]. With NIR light illumination, temperature-responsive behaviors of the stomatocyte nanomotors could be triggered. Furthermore, the nanomotors can move directionally toward H_2_O_2_ released from cancer cells, resulting in photothermal ablation effect of the cancer cells (Fig. 9b). The work demonstrates the feasibility of nanomotors-based system for effective photothermal cancer therapy.

## Conclusion and Outlook

The developments of micro/nanomotors have provided endless opportunities for intelligent cancer diagnosis and treatment. Micro/nanomotors have been widely used for cancer nanomedicine, as evidenced by breakthroughs currently made in this area. This review is mainly focused on the exploration and recent progresses of micro/nanomotors from three aspects: cancer-targeted delivery, diagnosis, and imaging-guided therapy. Utilization of the self-propelled ability of micro/nanomotors for addressing challenging issues raised by cancer research has promoted the development of novel delivery system, diagnosis methods, imaging probes, and so on, showing an encouraging potential for clinical cancer nanomedicine. Despite these advances, the application of micro/nanomotors in these aspects is still confronting multiple limitations and challenges.

For cancer-targeted delivery, the application scope of the micro/nanomotors-based delivery systems reported is still limited at the cellular level and in vitro research. Most of the micro/nanorobots reported are composed of inorganic materials. Although most materials are considered to be biocompatible and biodegradable, they may still be severely immunogenic and have maximum tolerance. Therefore, synthesized micro/nanomotors with versatility and excellent motion control in vivo are desired to provide new opportunities for efficient delivery in complex in vivo systems. In addition, real-time observation of the path of motor transport is expected to facilitate drug delivery to the target site, which will improve the transport efficiency. There are reports on the use of micro/nanorobots to achieve drug delivery in the stomach of mice, which also provide a guide for targeted drug delivery of tumors.

At present, micro/nanomotors-based detection methods mainly achieved by means of the change of speed or fluorescence single, which may encounter the problem of insufficient detection signals. Therefore, more attention should be paid to design multifunctional micro/nanomotors for the development of versatile detection technologies. There is significant potential to combine micro/nanorobots with other novel materials to fabricate versatile nanomachines in order to meet the needs of detection in complex environments. For example, micro/nanomotors combined with QDs or magnetic nanomaterials may open up new avenues for the construction of detection bioprobes and would greatly facilitate biodetection.

To date, only a few motors have been used for in vivo imaging and therapy because existing micro/nanomotor-based platforms are inefficient for deep tissue imaging and manipulation in vivo. For the perspective of clinical transformation, there is still a challenge to combine the clinically approved agents such as phospholipids, albumin, and PEG to prepare biocompatible micro/nanorobots. In addition, the design of comprehensive micro/nanomotors that integrate multiple imaging technologies with treatments may be particularly useful for combined therapy or image-guided therapy. Moreover, there is a need to develop micro/nanomotors driven by near-infrared light, which will provide potential possibilities for oncology applications in vivo.

Taken together, although some inspiring progresses have been made, there is still a long way to go from in vitro research to in vivo application. We hope this review will stimulate the further development of micro/nanorobots in the field of cancer nanomedicine and further benefit clinical cancer research.
